# “If you are on duty, you may be afraid to come out to attend to a person”: fear of crime and security challenges in maternal acute care in Nigeria from a realist perspective

**DOI:** 10.1186/s12913-020-05747-9

**Published:** 2020-09-29

**Authors:** Enyi Etiaba, Ana Manzano, Uju Agbawodikeizu, Udochukwu Ogu, Bassey Ebenso, Benjamin Uzochukwu, Obinna Onwujekwe, Nkoli Ezumah, Tolib Mirzoev

**Affiliations:** 1 Department of Health Administration and Management, Faculty of Health Sciences and Technology, College of Medicine, University of Nigeria, Enugu Campus, Enugu, Nigeria; 2 Health Policy Research Group, Department of Pharmaco-therapeutics, College of Medicine, University of Nigeria, Enugu Campus, Enugu, Nigeria; 3 School of Sociology and Social Policy, University of Leeds, 11.20 Social Sciences Building, Leeds, UK; 4 Department of Social Work, Faculty of Social Sciences, University of Nigeria, Nsukka, Nigeria; 5 Nuffield Centre for International Health and Development, Leeds Institute of Health Sciences, University of Leeds, Level 10 Worsley Building, Clarendon Way, Leeds, LS2 9NL UK; 6 Department of Community Medicine, Faculty of Medical Sciences, College of Medicine, University of Nigeria, Enugu Campus, Enugu, Nigeria

**Keywords:** Security, Safety, Primary care facility, Maternal and child health services, Providers, Users, Realist evaluation

## Abstract

**Background:**

Maternal and Child Health is a global priority. Access and utilization of facility-based health services remain a challenge in low and middle-income countries. Evidence on barriers to providing and accessing services omits information on the role of security within facilities. This paper explores the role of security in the provision and use of maternal health services in primary healthcare facilities in Nigeria.

**Methods:**

Study was carried out in Anambra state, Nigeria. Qualitative data were initially collected from 35 in-depth interviews and 24 focus groups with purposively identified key informants. Information gathered was used to build a programme theory that was tested with another round of interviews (17) and focus group (4) discussions. Data analysis and reporting were based on the Context-Mechanism-Outcome heuristic of Realist Evaluation methodology.

**Results:**

The presence of a male security guard in the facility was the most important security factor that facilitated provision and uptake of services. Others include perimeter fencing, lighting and staff accommodation. Lack of these components constrained provision and use of services, by impacting on behaviour of staff and patients. Security concerns of facility staff who did not feel safe to let in people into unguarded facilities, mirrored those of pregnant women who did not utilize health facilities because of fear of not being let in and attended to by facility staff.

**Conclusion:**

Health facility security should be key consideration in programme planning, to avert staff and women’s fear of crime which currently constrains provision and use of maternal healthcare at health facilities.

## Background

The high maternal and child mortality rates in different low and middle-income countries (LMICs), including Nigeria, have been attributed to inadequate utilization of facility-based maternal health care (MCH) services [1–3], given the direct association between utilization of maternal and child health services and improved health outcomes [4–6]. Studies have reported demand and supply-side barriers to accessing and utilizing facility-based interventions. Demand-side determinants of access and use of MCH services in different LMICs are reported to be influenced by individual, household, community and other contextual factors; with a variation across and within cultures [7–10]. Supply-side factors are attributed to inadequate number of health workers, reduced capacity and poor motivation of available staff, as well as other factors affecting their retention [6, 11]. Other supply side factors which impact utilization of facility services include medical supplies, drugs, equipment and infrastructure [12].

Although some studies have reported women’s feeling of security and safety while accessing facility services [13], only a few have actually addressed the potential challenges that can arise when facilities are perceived as secure or insecure by both providers and users, especially for vulnerable pregnant women, children and female health workers [14, 15]. This paper intends to contribute towards addressing this gap in the literature.

The Nigerian government, in 2012, through an MCH programme-Subsidy Reinvestment and Empowerment Program for MCH (SURE-P/MCH), embarked on improving supply and demand of MCH services. The supply component included recruiting, training and deployment of midwives and community health extension workers (CHEWs) to selected facilities, upgrading the facility infrastructure and supply of drugs and other consumables for MCH services. The demand component incentivised women in the community to access services through engaging another group of community health workers; the village health workers (VHWs), who sensitized and mobilized women within the community and encouraged them to access and use facility services [12]. This programme was targeted at the rural and underserved population throughout the country where most of the facilities and communities lack basic amenities.e.g. electricity, and the programme aimed to provide 24 h service, so night time security was important. However, there were no explicit measures in the programme design to ensure that the facilities were secured and that both providers and users of services felt safe while giving and receiving services, especially at night. As we explain later the issue of facility security emerged as an important aspect of both the provision and utilisation of health services. In Anambra state, where the study was carried out, increased crime rates have been reported to generally affect delivery of good governance [16].

Security has been defined as “pursuit of freedom from threats including violence” [17]. In this paper, we focus on how security operates at the micro (individual) and meso (institutional) context levels, hence, how this notion of protection against violent attacks or coercion and overall feeling of organisation safety causes staff and service users to act, and how these actions then affect healthcare access and uptake [18]. We drew on a complementary body of interdisciplinary theories of fear of crime and gender which determine perceived security by health workers and patients [19–21]. These incorporate structural, political and socio-economic factors reflecting a micro-meso analytical framework (Pain, 2000). The academic literature highlights four main perspectives that have characterized the approaches to studying the fear of crime. First is the relationship between fear of crime and social identity characteristics, such as age and gender. The second is the relationship between fear and structural factors like the physical layout of buildings. The third and fourth include the role of media, trust and informal networks [22]. The gender perspective and structural factors are relevant to our study because there is a clear gendered contextual component in the programme evaluated: female facility health workers (providers) on the supply-side, female VHWs and female users of MCH services (consumers) on the demand side. Fear of crime is recognized to be a significant problem, sometimes more than the crime itself and has also been linked to women’s subordinate social, economic and political status [23].

Crime prevention behaviours can be broken down into two major categories: avoidance and risk management strategies. Avoiding dangerous settings reduces the risk of running into potentially threatening situations. Risk management practices are used when one finds oneself in a dangerous situation and/or location and takes precautionary measures to be a less suitable target for victimization [23, 24]. These concepts, though mostly originating from higher-income countries, support transferable principles to low and middle-income contexts, where women are even perceived as more vulnerable and of comparably lower socioeconomic status, especially in the rural areas [14, 15, 25, 26].

This paper explores the role of health facility security and feeling of safety, which determine the provision and uptake of health services respectively, from the perspective of health staff, who were all females and health service users (pregnant women). It presents a component of a larger Realist Evaluation study described elsewhere [27, 28], specifically focusing on exploring the notion of facility security using qualitative data. In this paper, we define security as i) the absence of fear of crime and the feeling of safety within health care facilities. These feelings were assumed to support the provision of 24-h services by health workers and in this way, improve users’ access and utilisation of these services.

## Methods

### Study setting

Nigeria is a coastal West African country and is comprised of 36 states and a federal capital territory. This evaluation was carried out in Anambra state, located in the south-eastern region. Anambra state was purposively chosen as a case study for in-depth understanding of the inquiry into SURE-P/MCH, because of the researchers’ longstanding engagements in the state. It had a total population of 4,453,964, and a female population of 2,059,844 in the 2006 population census [29]. The potential economic drivers in the state are agriculture (farming, fishery, pasturing and animal husbandry), markets (trade and commerce), transportation (good road networks), natural resources and numerous industries. Anambra state has the largest number of women of child- bearing age and also has the largest number of literate women in the south east zone. There is high uptake of MCH services in the state,although this is predominantly from private facilities [30]. Although there is no insurgency in Anambra state, unlike in some other states in the country, there is however, reported high rates and a general perception that security is a major challenge affecting delivery of good governance in the state, [16, 31].

The SURE-P/MCH programme was initially carried out in 12 primary health care (PHC) facilities, beginning from October 2012. A year later, another 12 PHCs were selected and included in the programme, which ended in November 2015. This study focused on the initial 12 facilities because they had a longer experience of the intervention. The following MCH interventions-antenatal care, facility- based delivery, post-natal care, immunisation and family planning were implemented in all 12 PHCs. Facility managers and health workers (nurses, midwives and community health extension workers) were all females. In addition, each facility was allocated six village health workers, all females, who identified pregnant women in the community and encouraged them to access and use facility based MCH services. Each facility also had a ward development committee (WDC) made up of a gender mix of 8–10 community members.

### Study design

This was a qualitative, exploratory case study using a Realist Evaluation (RE) approach [32, 33]. RE moves beyond cause and effect, to focus on ‘what works, how it works, under what conditions and for whom it works’ using the context, mechanism and outcome (C-M-O) configurations as a heuristic [27, 32, 34]. Context refers to the conditions in which programmes are introduced, and can include political and economic conditions, cultural norms and beliefs. Mechanism includes two aspects, first the process of reasoning of how subjects interpret and act upon programme intervention, at a given time, in a given context, and secondly how they interact with available programme resources. Outcomes are described as the patterns of intended and unintended consequences that result from mechanisms triggered in different contexts, and may be proximal, intermediate or distal [32, 35]. Theories about how programmes are expected to work (“programme theories”) are developed based on this configuration and are then iteratively tested and refined with empirical data gathered through appropriate methods and triangulated with available literature [32, 36]. The overall evaluation approach sought to answer the question ‘what works for whom under what circumstances, how and why’ using qualitative methods. Study was carried out in two phases. Phase 1 (P1) was exploratory and was based on two initial working theories (one Supply and one Demand), largely built from relevant contextual literature and logic map development [28], about how programme interventions introduced into a given context will trigger mechanisms which are acted upon to result in observed or implied outcomes. The Supply side theory which incorporates the state of the health facilities is as follows; ***“****In the context of irregular payment of salaries and poorly functioning facilities in Anambra state* ***(C),*** *if different incentives (e.g. regular payments, training and improved working environment) are provided in a timely manner, then these interventions will make health workers feel motivated* ***(M),*** *and lead to sustained performance, job satisfaction and improved retention of staff* ***(O).”*** We conducted in-depth interviews to glean initial knowledge to build our programme theories. Security emerged as a distinct theme from these exploratory interviews and formed a distinct programme theory, which was then iteratively tested, validated and refined in phase 2 (P2), based on views of patients and health staff. We report our findings from both phases (P1 and P2) using C-M-O linkages.

### Sampling and data collection

In Phase 1 (March–October 2016), eight health facilities were sampled to include the four facilities that had the full complement of programme components (additional demand side intervention, although not a focus of this paper) and another four chosen randomly. Interview respondents were purposively selected from these facilities to include the facility managers, a programme midwife and a pre-existing (before programme) health worker and the VHWs. On the demand side, we sampled service users (women who had received maternal care services during the programme intervention (October 2012–November 2015, and were also receiving maternal and child care services during the study period), but who did not necessarily need to be pregnant at the time of the interview. As a result, most of the participants were multiparous (more than one facility delivery). We also sampled WDC members, who are community representatives that oversee the functioning of the facilities. Programme managers and relevant state and local government level policymakers were also interviewed. We also visited the 12 health facilities during both phases of data collection, for direct observation of the structural security components (perimeter fencing, secure gates, security guards and staff accommodation).

Data collection included document reviews to ascertain the programme’s approach, in-depth interviews (IDIs) and focus group discussions (FGDs) to explore the views and experiences of a diverse group of stakeholders.35 in-depth interviews (IDIs) were conducted with policymakers (*n* = 9), programme managers (*n* = 10), facility managers (*n* = 8) and facility health workers (n = 8). FGDs were conducted with eight groups of service users (8–10 respondents per group), eight groups of VHWs (6 respondents per group) and eight groups of WDCs (6–8 respondents per group). Health workers comprised nurses, midwives and community health extension workers (CHEWs). Researchers, who were trained in realist qualitative interviewing [33], conducted all interviews. Information gleaned from these interviews were synthesised and informed our programme theory on Security and Safety. In Phase 2(July–December 2018), to test our middle range programme theory which we had built from information gathered from Phase 1, further interviews were conducted. These included 17 IDIs with facility health workers (*n* = 8), VHWs (*n* = 9) and FGDs with four groups of service users (5–10 per group) because these were the respondent groups (providers and users) directly involved with providing and utilizing round the clock facility-based MCH services. Although the VHWs were not officially scheduled for night duties, their perception on security, in their interphase role, was explored through IDIs in Phase 2 to further explore their experiences in depth. One of the four FGD groups with service users had 5 participants although 10 women had accepted to participate. A heavy rain on the day of scheduled interview constrained their attendance due to poor road access. The other three FGD groups had 8, 8 and 10 participants respectively. In spite of this constraint, researchers noted that saturation was achieved when compared with the other FGD interviews In addition, there is also evidence in the literature that favours small number (3–5) of participants for FGDs, as this has greater potential to explore complex topics in-depth, while there are also arguments for medium (6–8) and large number (6–12) participants to capture a wider range of views. It is customary to present focus group size in ranges in protocols because of the uncertainty of how many participants will be able to attend on the day. This uncertainty increases when participants are from poorer and undeserved backgrounds which can be particularly affected by unexpected contingencies [37–39]. All Phase 2 interviews were conducted with different respondents (from Phase 1 respondents) but within the study population (providers and users of services in the facilities that had received the SURE-P/MCH intervention). This was in order to validate and further refine our programme theory built from Phase 1 interviews. In each phase, respondents were asked to retrospectively reflect about the programme intervention from their perspective. All interviews lasted between 40 and 60 min and researchers reached saturation. Interview question guides for various groups of respondents were developed for this project and they are included as Supplementary files.

### Data analysis

Interviews were transcribed verbatim and analysed. Manual thematic analysis was undertaken, systematically identifying emerging themes, which were then organised according to whether they were perceived to be Context, Mechanism or Outcomes and initial linkages between all these were recorded. Each transcript was coded by two researchers, initially individually and then came together to agree on any disparities. Further quality check was conducted on randomly selected transcripts by two other researchers. Data analysis was guided by our hypothesis that the presence or absence of security components given (by the programme) and/or existing resources and how various stakeholders (service users and providers) interacted with these resources to produce behaviours which manifest in their actions. It is these combinations that give rise to the outcome patterns observed and reported here. With information synthesised from Phase 1, we proposed a programme theory for Security and Safety as follows; *“In the context where programmes or communities ensure employment of security guards, erect perimeter fences and there is availability of accommodation and adequate lighting in the health facility premises, health workers and service users are likely to feel safer and therefore willing to provide and use round the clock MCH services, leading to improved access and utilization of MCH services*.” We tested this theory, by retroductively analysing information gathered from the Phase 2 interviews. We then reported varying explanatory CMO configurations which emerged from our data, in line with the RAMESES Realist Evaluation reporting standards [40]. In the analysis, we acknowledge that realist evaluation is not primarily concerned with whether secure facilities (or insecure facilities) directly lead to increased (or decreased) facility access and utilization (i.e. causation), rather we used RE to explore how participants interacted with the resources (emotional, social, material, economic and sometimes political) offered by the SURE-P/MCH programme to produce actions which led to observed programme outcomes [34].

### Ethical considerations

Ethical approvals were granted by the School of Medicine Research Ethics Committee at the Faculty of Medicine and Health at the University of Leeds (ref: SoMREC/14/097) and the Health Research Ethics Committee at the University of Nigeria Teaching Hospital (ref: NHREC/05/02/2008B-FWA00002458-1RB00002323). Written informed consent was obtained from all study participants and they were assured of confidentiality during reporting of findings.

## Results

One or more contextual factors were identified within the programme intervention which may or may not interact to trigger mechanisms, which causes service providers and users to behave in ways that lead to various outcomes. Below, we present different C-M-O configurations which explore the presence or absence of security components during the programme, and community efforts to maintain facility security after the programme. We present three revised CMO configurations (after testing our initial programme theory) related to the presence or absence of security in the healthcare facility:

CMO1: *Provision of perimeter fence, security guard, adequate lighting and staff accommodation within the health facility supports health workers and service users to feel safe and confident to provide and use 24- h services.*

These security components were in place, in different combinations, in some study facilities and not in others. In facilities where they were in place or had been instituted at the beginning of the programme, health workers and service users perceived the facilities as guarded and secure, hence felt safe to provide and use services. This was reported by programme managers, users and staff and exemplified with the following quotes,

*“It (security) was a big problem. That was why they could not run 24-hours services. So, we had to fence some of the health centers and put [i.e. recruit] security men, just to make sure that they are secured … So, if they [health workers] get there, number one, security is utmost to them. It is very important that at least you ensure that their own lives are safe so that they can save other women’s lives.” (P1-IDI, programme manager, male).*

When the programme instituted some of these security components, there was a feeling of safety,

*“Let me add to that. There was a time when this place was not fenced and there was no security man. It was when SURE-P started that the community came together and decided to fence this place and employed a security man that the villagers started feeling comfortable to stay here after delivery for days”. (P1-FGD, VHWs****).***

Availability of staff accommodation within the health facility resulted in more health workers living within the facility, thus making health workers feel safe at night, being aware that other co-workers (and their families) were living in the facility. This increased the provision of 24- h services, and utilization especially at nights, which explained how security personnel and healthcare staff were available during nocturnal obstetric emergencies:

*“There was a woman around my house who my husband called when I was in labor, she ran out to get a taxi. We got here by 2am and the gateman went to get a nurse and they immediately attended to me they took very good care of me****!”*** *(P2-FGD-, female, farmer/petty trader).*

During the programme, health facilities had adequate numbers of staff that made it possible for there to be more than one health worker running a shift. The fact that they worked in pairs made the health workers feel safer, in addition to the feeling that they had help at hand from other staff living within the facility accommodation, if there was any threat,

*“In a shift, we may have up to two or three [staff], so being that you have a colleague, it will still stimulate you, despite the fact that there is no gate or fence, but having someone you are working with, that will scare away the fear. So, it really helped us and increased the health care services that we gave to them****.”*** *(P2-IDI, health worker-CHEW).*

In opposition to this, the absence of security in healthcare facilities was identified as having a different impact which was phrased as:

CMO2: *Absence of security (fence, security guard and adequate lighting) and no staff accommodation within facility made health workers feel unsafe within the facility, especially at night, with a resultant reduction in 24 h access and utilization of facility services by service users.*

Before the programme, some facilities were not fenced and did not have gates and security men to safeguard health workers and patients. This created a feeling of fear and insecurity among the health workers and service users, which resulted in challenges highlighted by the participants. In some facilities where night-time security was not assured, health workers resorted to locking the facility doors at night and would not respond when potential service users knocked as they were not certain they were free from threats. Pregnant women and their family members experienced being turned back at unguarded facility doors from within because the night duty staff were unable to ascertain the identity of the potential service users and did not feel safe to open the doors.

“*… .. many centers don’t have security men. We have about 40 health facilities here, both full-fledged primary health care and health posts, but we have only one security man. Because most deliveries are usually in the night, when these women come shouting, crying … … .and there is no security man to help assure that the person that has come is actually a pregnant woman and not a robber, the nurse will not come out …*” *(P1-IDI, Local Govt. Policymaker-male).*

Even when women had available emergency transportation, after arrival at the premises, delayed or denied facility access were reported by users and confirmed by staff who feared for their own safety. Once a decision to seek medical care has been made, other obstacles had to be overcome when the medical facility was reached. These included: delay in receiving prompt care after reaching the hospital, and if access was denied due to the fear of crime, women had to seek care at another medical facility. Another quote from a VHW expressed this same concern below:

*“The nurses were scared for lack of security man then, and the women started reporting to us that some women in labour had gone there at night, and they stayed and stayed without seeing anybody, so they left to another hospital. So, it posed a very big challenge then.” (P2-IDI−/VHW).*

Within the context of these pre-existing conditions, when the programme resources were phased out some communities collaborated to preserve security in the health centres ensuring the sustainability of this outcome. This was phrased as:

CMO 3: *In facilities where the community ensured sustained presence of security guard after SURE-P/MCH programme, health workers continued to feel safe and confident to provide 24 h services, and hence sustained, improved service delivery and utilization.*

Participants highlighted the collaborative efforts made by their communities to safeguard the PHCs and how these efforts produced positive effects including some communities employing and paying security guards to help secure the facilities and medicines that were brought therein. Fences, gates and particularly, security personnel encouraged the health workers to feel safe in their places of work and had confidence in their host communities, which also encouraged community members to utilize PHC facilities.

*“There was a time when people’s children were stolen [abducted]. The community decided to stop this through employing a security man who will safeguard the facility. The security guard was paid by the Ward Development Committee (WDC)” (P1-FGD, male WDC member).*

Conversely, in communities that were unable to sustain the programme inputs or take initiatives to provide security, service provision and utilization became constrained once the programme ended. An example is illustrated with the quote below:

*“A friend of mine who was pregnant came to the health center with her husband in the middle of the night without knowing that the programme had ended. We stood long at the gate and knocked for almost an hour but there was no response. I went to the second gate and it was empty, I got tired and picked a stone and threw it on the roof. Someone eventually came out …*” *(P2-FGD-, female, petty trader).*

A service provider also noted as follows,

*The reason is that in this facility there is no fence, few months ago, the sumo (water pump) that we were using to pump water from the borehole was stolen from this place, and we were all thanking God that they did not penetrate into our homes here … we need light, generator, SURE-P used to supply things like that but since SURE-P left, the one we had is no longer functioning very well now” (P2-IDI, health worker/CHEW).*

CMO 4: *Presence of a male security guard in the facility made the female health workers feel safer and more secure and confident to deliver 24 h services leading to improved service delivery, access and utilization*.

Of all the security components outlined by respondents, the presence of a male security guard at night appeared to be the most important. Presence of a gate at a health facility was undermined by lack of a security personnel to guard the gate and this was also a source of anxiety,

*“We didn’t have a security man, though there was a gate which we lock ourselves, but when the women come at night, they call us because our door is somehow close to the gate, so when they call us, we open the gate for them … ..It was scary” (P2-IDI, HW/female Midwife).*

In Nigeria, the responsibility of performing security roles such as opening gates for and screening visitors at night usually falls on male security guards. Where there were no security guards employed, and the health workers had to carry out this function at nights, it made them feel vulnerable and sometimes they completely refused to take on these roles. Staff believed that having a male security guard in their midst was a source of strength for them.

*“A woman is not supposed to open this gate for a visitor in the night, a woman, a nurse. It is the watch night [security guard] that will open it, come to the quarters [accommodation] and call the nurse whether there is a fence or not. It is the watch night that will call us before we come out. At least we have hope that we have a man in our midst. Nurses are women but all the time we will be the watch night, we will be the nurse, we will be everything and one person on duty. … Security matters a lot* ***…***” *(P1-IDI, facility manager/health worker, female).*

The added concern and feeling of insecurity at nights is also buttressed with the following quote,

“*There was a security man during the SURE-P period. There was a man sent by the local government that they used to pay. He stayed mostly at nights, we used to tell him to leave in the morning that we need him more in the nights.” (P2-IDI-VHW).*

Staff also pointed out that service users also felt safer once they arrive at the facility. Although the gender of the security guard and staff was identified by respondents as important, there is a more nuanced understanding of the security guards in this context, not only in terms of gender but also on their knowledge of the community and ability to identify strangers who may be perceived as a threat, conflict management skills experience, crime deterrence equipment and symbolic authority (i.e. uniform).

In summary, these CMO configurations show that a well-resourced MCH programme (what) will be beneficial (work) to MCH service providers and users (Whom), in circumstances where there are adequate resources and the health facility is secure enough (especially at night) to make them feel safe to offer and use services (how). This could be because, anecdotally, a large proportion of deliveries occur at night (why). We further refined our programme theory as follows, *“In the context where programmes, or communities ensure* ***sustained*** *employment of male security guards, erect perimeter fences and there is availability of accommodation and adequate lighting in the health facility premises,* ***female*** *health workers and service* ***users (pregnant women)*** *are likely to feel safer* ***(especially at night)*** *and therefore willing to provide and/or use MCH services respectively, thus ensuring the provision of round the clock MCH services, and improved access and utilization of MCH services.”* We further acknowledge that this theory can and will be refined by future studies.

## Discussion

This paper identified the decisions and behaviours triggered in female health workers and service users, by the presence or lack of adequate facility security components and elaborated the importance of health facility security in the provision and uptake of MCH services at grassroots level [18]. The findings highlight two key issues. The first is that, different security elements as operationalised in this study (fencing, gate, security guard, electricity, staff living within facility accommodation and more than one staff running a shift) work in different combinations to contribute to perceived feelings of safety, depending on whether these components are available or not. Second, the study highlights how an otherwise well-resourced programme can be constrained or facilitated by a singular component (in this case security), where it is included or excluded in the initial programme design or during implementation.

Experiences of healthcare workers, who were all females, in the study area, differed according to whether they perceived their facilities to be safe or unsafe to provide 24-h services. Our study found that in facilities where a combination of the presence of security guard and structural security (perimeter fencing, adequate lighting) were in place, the health workers and service users felt safe to provide and utilize facility-based MCH services respectively, and expectedly, the reverse was the case in perceived insecure facilities. Most important of these, was the sheer physical presence of a male security guard which made health workers feel safer within the facilities.

One action the health workers took to ensure their safety in the facilities at night was to lock the facility and not let anyone in, through the night. Even when users were willing to initiate healthcare utilization, healthcare staff prevented utilization from happening by physically closing the access to the facilities which potentially could increase the risk of negative healthcare outcomes for mothers and newborns. The desire to utilize proven, effective maternal and child healthcare (MCH) services available in primary health care (PHC) facilities in any context will depend on the value mothers place on the primary health care services and their estimation of the goal of achieving good health for their children. Household, community and other contextual factors, like previous or long-term experiences e.g. mothers with negative experiences from the health facilities in the form of lack of health personnel, poor attitude of the health workers, etc. will place lower values on the use of health care facilities [3]. Service users who have been turned away at night by health workers may perceive the facility as insensitive, unsafe or inadequate for their MCH needs and hence the perception may trigger mechanisms that will likely alter their health-seeking behavior. Health workers’ attitude in guarding their own safety may result in negative consequences such as complications during labor, including maternal or child death, depending on the stage at which a woman attends the facility. In the longer term, this may make women not to even access the facility at all and seek alternative care from other sources, such as traditional birth attendants [41], some of whom may not be skilled [42, 43].

Lack of security in facilities (absence of perimeter fencing, inadequate lighting, lack of within-facility accommodation and absence of security guards) have raised issues of access across some other LMICs (Tanzania, Nepal, Swaziland, South Africa and Kenya) to varying degrees, especially in the rural areas. These range from the limitation of provision and access to services, to service providers changing jobs to facilities where they perceive to be more secure [14, 15, 25, 26, 44]. Low retention of public sector nurses in South Africa has been related to lack of security in the public facilities [25]. Concerns about different crimes; stolen babies, threatening of staff have been documented in facilities in some areas of South Africa and in those areas, nurses were three times more likely to experience crime and violence in the workplace than other occupational groups [45, 46].

Security components also need to be provided in an acceptable combination to providers and users of facility services, in order to trigger the feeling of safety. Of these, however, the presence of a trained male security guard within the facility is perceived to be crucial to the feeling of safety by female health workers. This supports theories of other underlying social factors to the fear of crime [47]. It would appear that provision of security guards, a pivotal security component, was left at the discretion of the community and not explicitly incorporated in the programme design either in the supply or demand side. Where the community took ownership of the programme and intervened to provide security guards, this led to improved provision and utilization of services. It has been argued, that community health worker (CHW) programmes, like the SURE-P/MCH, while being integrated into the health sector also need to be embedded in the community systems, for a more holistic approach to service delivery, and that this is required in order to successfully implement CHW programmes at scale as has been experienced in some LMICs [48]. However, there is also another significant factor related to health workers’ safety. The presence of other health workers living within the premises, some with their families, increased the perception that there were other people within reach, in case of any threats. This is a situation where structural modifications lead to ‘increased ownership of space and social interaction’ as theorized by Koskela and Pain [49]. Lack of staff accommodation was cited as one of the reasons why health workers did not want to be posted to work in remote areas of Nepal [26].

There is ample anecdotal evidence across the data from respondents that most deliveries take place at night. This contributed to a heightened sense of vulnerability and feeling of unsafety amongst service providers and users alike. Darkness and being alone are two potential factors that heighten the fear of personal safety and indicate potential risk [50]. Some of the study facilities did not have electricity, with the result that the facilities were dark at night and this resulted in health workers not providing needed services, and service users seeking healthcare elsewhere [43].

The strengths of this study include the long- term duration (five years) of this realist evaluation which allowed sufficient time to develop and subsequently test, validate and refine our programme theories. Limitations are the relatively small scale of the study, being carried out in only one state, hence not allowing for comparison of different state contexts and their influence on programme outcomes. The study also focused on health facility service users and so cannot be generalised to non-facility users since they may be systematically different from the users. Further studies also need to specifically disaggregate health facility services, especially deliveries, by time of day, to lend evidence for policy recommendations. Finally, the second round of interviews in 2018 may have been more prone to recall bias but this was controlled with the iterative nature of the realist interviews and that researchers achieved saturation. We also note that in complex interventions such as this, positive or negative outcomes cannot be attributed to one programme resource alone, hence the observed CMO configurations from this study are not reported as causal linkages [51].

## Conclusions and recommendations

MCH programmes, can be constrained or facilitated by a singular component (in this case security), if it is included or excluded in the initial programme design or during implementation. In some LMIC countries, workplace safety is severely compromised and staff fear and reality of violence in healthcare facilities may constrain intended programme outcomes. Although socio-cultural beliefs and individual barriers are often the focus of investigations of factors that influence women’s likelihood to seek healthcare during pregnancy and labour, our findings highlight the importance of structural factors including the provision of adequate physical as well as human security elements in health facilities to improve access to grassroots-level MCH services. We believe these findings are transferable principles especially to other LMICs. Hence, facility-based health programmes need to explicitly incorporate security of service providers and users in programme designs.

Federal and state governments need to work closely with Local Government authorities and facility managers to ensure that the security needs of PHCs are met and workplace safety is ensured [52]. Communities should be incentivized by the government to enable them to employ community members as security personnel for the facilities, as they stand a better chance of knowing how, and from whom to protect the facility.

## Data Availability

Data supporting our results will be uploaded on to the University of Leeds Repository (http://archive.researchdata.leeds.ac.uk/) by July 31 2020. Before this date, all data can also be made available on request from the corresponding author.

## Abbreviations

CMO: Context-Mechanism-Outcome
CHW: Community Health Workers
CHEW: Community Health Extension Worker
FGD: Focus Group Discussion
IDI: In Depth Interview
LMIC: Low-Middle Income Country
MCH: Maternal and Child Health
PHC: Primary Health Care
RAMESES: Reaist And Meta-Narrative Evidence Synthesis: Evolving Standards
RE: Realist Evaluation
SURE-P: Subsidy Reinvestment and Empowerment Programme
VHW: Village Health Worker
WDC: Ward Developmemt Committee
